# Prognostic value of SS18–SSX fusion type in synovial sarcoma; systematic review and meta-analysis

**DOI:** 10.1186/s40064-015-1168-3

**Published:** 2015-07-25

**Authors:** Tadahiko Kubo, Shoji Shimose, Jun Fujimori, Taisuke Furuta, Mitsuo Ochi

**Affiliations:** Department of Orthopaedic Surgery, Graduate School of Biomedical Sciences, Hiroshima University, 1-2-3 Kasumi, Minami-ku, Hiroshima, 734-8551 Japan

**Keywords:** Meta-analysis, Synovial sarcoma, Fusion gene, SS18–SSX, Survival

## Abstract

SS18–SSX (formerly called SYT–SSX) fusion gene has been established clinically as a molecular diagnostic test for synovial sarcoma, but the prognostic value of the fusion gene variant for survival is controversial. The objective of this systematic review is to provide an up-to-date and unprecedented summary of the prognostic impact of SS18–SSX fusion type in synovial sarcoma. Studies evaluating SS18–SSX fusion type as a prognostic marker in synovial sarcoma were systematically searched for in MEDLINE, EMBASE, and Web of Science. Comparative analysis of the pooled hazard ratios (HR) between fusion types was carried out, in order to assess the likelihood of overall survival (OS), disease-specific survival (DSS), progression-free survival (PFS), and metastasis-free survival (MFS). A total of 10 studies comprising 902 patients with synovial sarcoma were considered for the meta-analysis. The pooled HR for eight eligible studies evaluating for OS or DSS was 1.28 (95% confidence interval: 0.81–2.00), suggesting no significant difference between SS18–SSX1 and SS18–SSX2 (P = 0.29). For seven studies which evaluated for PFS or MFS, the presence of SS18–SSX1 may indicate a lower survival probability than that of SS18–SSX2, although the effect did not reach a level of statistical significance (P = 0.09). There was no significant difference in OS or DSS between SS18–SSX1 and SS18–SSX2, but there were indications of SS18–SSX1 being an unfavorable prognostic factor of PFS or MFS. Further studies including cohorts with a longer follow-up period are needed.

**Electronic supplementary material:**

The online version of this article (doi:10.1186/s40064-015-1168-3) contains supplementary material, which is available to authorized users.

## Background

Synovial sarcoma, accounting for 7–10% of all soft tissue sarcoma, most commonly occurs in the extremities of young adults. Recent therapeutic progress in surgery, chemotherapy, and radiotherapy has improved the prognosis of survival, with a 5-year overall survival rate of 50–80%, and several prognostic factors being reported such as patient’s age, tumor size, and histological grade (Lewis et al. 2000; Spillane et al. 2000; Bergh et al. 1999). A recurrent chromosomal translocation, t(X;18)(p11.2;q11.2), fuses the SS18 (formerly called SYT) gene on chromosome 18 to SSX1, SSX2, or, rarely, SSX4 on the X chromosome (Clark et al. 1994). Since SS18–SSX (formerly called SYT–SSX) fusion gene can be found in more than 95% of synovial sarcoma by reverse transcription-polymerase chain reaction or fluorescent in situ hybridization, it is considered to be an established clinically diagnostic marker for this type of tumor. Moreover, this translocation is regarded as a chimeric fusion oncogene in the development of synovial sarcoma.

The extent of the prognostic significance of SS18T–SSX fusion gene variant remains unclear. The first small report by Kawai et al. (1998) statistically showed that patients with tumors bearing SS18–SSX2 had a better metastasis-free survival (MFS) rate than those with SS18–SSX1. A large study reported by the same institute did not proved a significant difference in overall survival (OS) for all patients, but showed that only for those without metastasis at diagnosis, suggesting that once a tumor metastasizes, this event outweighs any prognostic influence of the fusion gene (Ladanyi et al. 2002). A multi-institutional study of 141 patients in Europe did not show any correlation between the fusion gene and the likelihood of any survival (Guillou et al. 2004). If the SS18–SSX fusion type is associated with survival outcomes, it should play an important role in tumor cell behavior, affecting its initiation, progression, and metastatic activity. However, the results of studies looking for a relationship between the fusion type and tumor cell proliferation are also controversial. Two studies have demonstrated a higher cell proliferation in SS18–SSX1 tumors (Skytting 2000; Inagaki et al. 2000). Conversely, Guillou et al.’s (2004) study showed that SS18–SSX1 tumors tended to be less necrotic than SS18–SSX2, but there was no difference in the degree of differentiation or mitotic activity between them.

The purpose of this systematic review is to provide an up-to-date and unprecedented summary of the prognostic value of SS18–SSX fusion type in synovial sarcoma. We used a systematic literature search and meta-analysis to compare the difference in survival rates of synovial sarcoma patients with SS18–SSX1 and those with SS18–SSX2.

## Methods

### Literature search

A systematic search was performed according to the Preferred Reporting Items for Systematic Reviews and Meta-Analyses (PRISMA) statement (Liberati et al. 2009). The main research question was defined using the Target Population, Index Test, Comparator Test, Outcome, and Study design (PICOS) strategy, which was formulated into a search query. A search based on a combination of the terms ‘‘synovial sarcoma’’, “survival”, and ‘‘SYT–SSX or SS18–SSX’’ was performed without a time search limitation, using the following three search engines: MEDLINE, EMBASE, and Web of Science.

### Study selection

Two reviewers (TK and TF) independently assessed potentially relevant articles for eligibility using predetermined criteria. The procedure to include or exclude articles was hierarchical and initially based on the study title, then on the study abstract, and finally on the full study article.

The inclusion criteria were: (1) original English articles; (2) to evaluate SS18–SSX fusion gene as a prognostic factor in synovial sarcoma; (3) sufficient raw data to estimate the log hazard ratio (logHR) and standard error (SE) for OS, disease-specific survival (DSS), progression-free survival (PFS) or MFS of patients with SS18–SSX1 compared with those with SS18–SSX2; (4) studies with a sample size of 30 or more. When subsets of data were reported in more than one article, the most recent article was chosen.

### Data extraction

The two investigators (TK and JF) independently reviewed the included articles and extracted the following information on time-to-event data: the hazard ratio (HR) and 95% confidence interval (95% CI), *P* values of the log-rank test and event numbers, or raw data of all patients. Then, the methods provided by Parmar et al. (1998) and Tierney et al. (2007) were used to convert such data into the logHR and SE.

### Quality assessment

The quality of study designs was evaluated using the Newcastle–Ottawa scale (NOS) for quality assessment of cohort studies (Stang 2010). A star system of the NOS has been developed for the evaluation, the highest value being nine stars.

### Meta-analysis

Meta-analysis was conducted using the generic inverse-variance method. Heterogeneity of the HR of each study was assessed by the inconsistency index I-square (I^2^) test as well as by the χ2 test. An I^2^ > 50% and/or *P* < 0.05 was considered statistically significant. A random effect model (Der Simonian and Laird method) was applied if heterogeneity was observed, while a fixed effect model was used in the absence of between-study heterogeneity (I^2^ < 50%, *P* > 0.05). Publication bias was estimated using funnel plot asymmetry tests. All meta-analysis was performed using Review Manager software, v. 5 (Cochrane Collaboration, Oxford, UK). *P* < 0.05 was defined as statistically significant.

## Results

### Literature search and selection of studies

**Fig. 1:**
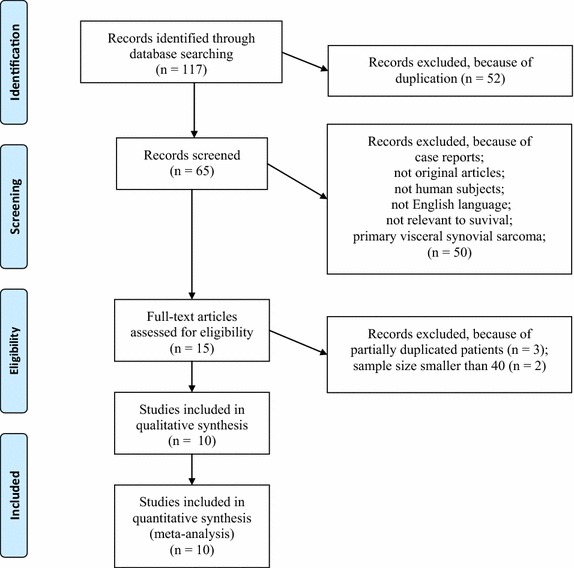
A flowchart of the article-selection process.

The main research question according to PICOS was P, patients with synovial sarcoma; I, the presence of SS18–SSX1 fusion gene; C, the presence of SS18–SSX2 fusion gene; O, OS, DSS, PFS and/or MFS; S, retrospective cohort studies. Using the predefined search strategy, we identified 117 potentially eligible articles, of which 52 were excluded due to duplication and 50 were excluded after reviewing the title and abstract. Finally, five studies were excluded after reviewing the complete article (Kawai et al. 1998; Skytting 2000; Nilsson et al. 1999; Hill et al. 2003; Canter et al. 2008). A total of 10 articles comprising 902 patients with synovial sarcoma fulfilled all of the inclusion criteria (Ladanyi et al. 2002; Guillou et al. 2004; Panagopoulos et al. 2001; Mezzelani et al. 2001; Takenaka et al. 2008; ten Heuvel et al. 2009; Sun et al. 2009; Krieg et al. 2011; Charbonneau et al. 2013; Ren et al. 2013). The detailed selection procedure in the meta-analysis is shown in Fig. 1 and Additional file 1.

### Study description and quality

Table 1 shows the principal characteristics of the 10 studies included in the meta-analysis. The overall quality of the included studies evaluated by the NOS assessment was adequate (mean 6.8 out of 9 stars) and there was no study with less than five stars. Low rating items were blindness of assessment and/or adequate follow-up period in the outcome categories (Table 2).

**Table 1:** Clinical characteristics of the patients included in the meta-analysis

References	Country	Study design	Total N	Median/mean follow-up
Panagopoulos et al. (2001)	Sweden	N/D	60	35 months
Mezzelani et al. (2001)	Italy	Consecutive	72	N/D
Ladanyi et al. (2002)	USA	Retrospective	242	2·7 years
Guillou et al. (2004)	Switzerland	Retrospective	165	37 months
Takenaka et al. (2008)	Japan	Retrospective	108	54 months
ten Heuvel et al. (2009)	Netherlands	N/D	45	55 months
Sun et al. (2009)	China	N/D	141	54 months
Krieg et al. (2011)	Switzerland	Retrospective	62	11.4 years
Charbonneau et al. (2013)	USA	Retrospective	103	N/D
Ren et al. (2013)	China	Retrospective	88	42·7 months

References	Analyzed N	Fusion gene analysis	SS18–SSX1/SS18–SSX2	Survival analysis
Panagopoulos et al. (2001)	47	PCR	31/16	DSS, MFS
Mezzelani et al. (2001)	64	PCR	40/24	MFS
Ladanyi et al. (2002)	202	PCR or FISH	122/80	OS
Guillou et al. (2004)	141	PCR	99/42	DSS, MFS
Takenaka et al. (2008)	91	PCR	57/34	OS, MFS
ten Heuvel et al. (2009)	45	PCR or FISH	27/18	DSS, MFS
Sun et al. (2009)	141	PCR	50/91	DSS, MFS
Krieg et al. (2011)	43	PCR	30/13	OS
Charbonneau et al. (2013)	40	PCR	24/16	PFS
Ren et al. (2013)	88	PCR	47/41	OS

N/D not document, N number of patients, PCR reverse transcription-polymerase chain reaction, FISH fluorescent in situ hybridization, OS overall survival, DSS disease-specific survival, PFS progression-free survival, MFS metastasis-free survival.

**Table 2:** Newcastle–ottawa quality assessment scale for cohort studies

References	Selection	Comparability	Outcome			Total score
			Assessment of outcome	Follow-up long enough for outcomes	Adequacy of follow-up of cohorts	
Panagopoulos et al. (2001)	4	2	0	0	0	6
Mezzelani et al. (2001)	4	2	0	0	1	7
Ladanyi et al. (2002)	4	2	1	0	1	8
Guillou et al. (2004)	4	2	1	0	1	8
Takenaka et al. (2008)	4	2	1	0	0	7
ten Heuvel et al. (2009)	4	2	0	0	0	6
Sun et al. (2009)	4	2	0	0	0	6
Krieg et al. (2011)	4	2	0	1	1	8
Charbonneau et al. (2013)	4	2	0	0	0	6
Ren et al. (2013)	4	2	0	0	0	6

### Meta-analysis

**Fig. 2:**
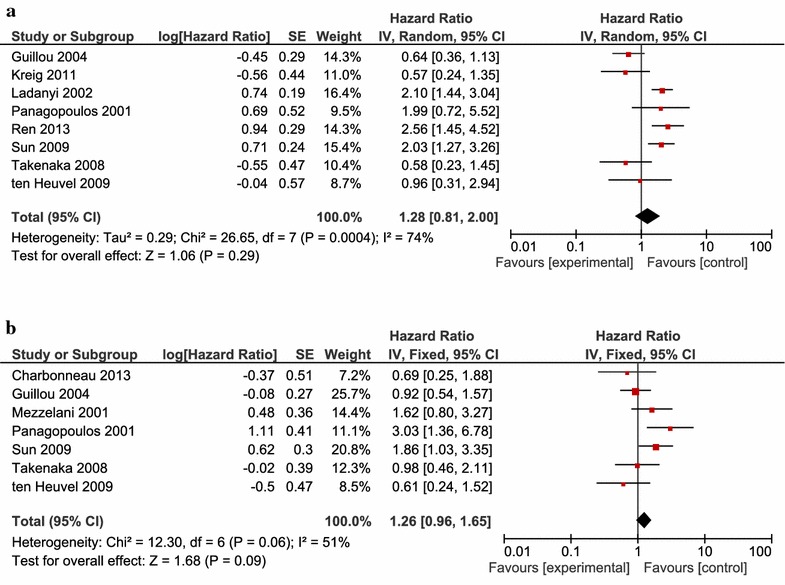
Forest plot of hazard ratios between SS18 and SSX fusion type for OS or DFS (**a**) and PFS or MFS (**b**). Square size of individual studies represents weight of study. Vertical lines represent 95% CI of pooled estimate. “Experimental” indicates patients with SS18–SSX1 fusion gene. “Control” indicates patients with SS18–SSX2 fusion gene.

DSS was analyzed together with OS to examine death-related survival. There was a significant heterogeneity among the included eight studies consisting of 798 patients (*P* = 0.0004, I^2^ = 74%); thus, the random effect m odel was used. Overall, the pooled HR for OS or DSS was 1.28 (95% CI 0.81–2.00), suggesting that no significant difference existed between patients with SS18–SSX1 and SS18–SSX2 (*P* = 0.29) (Fig. 2a).

The analysis of PFS and MFS was combined to examine disease-free survival. For seven studies composed of 569 patients, a pooled HR and its 95% CI were calculated with the fixed effect model because of the mild heterogeneity among the studies (*P* = 0.06, I^2^ = 51%). The result showed that SS18–SSX1 may predict poorer PFS or MFS than SS18–SSX2, and that the pooled HR was 1.26 (95% CI 0.96–1.65), although the effect did not reach the level of statistical significance (*P* = 0.09) (Fig. 2b).

### Publication bias

**Fig. 3:**
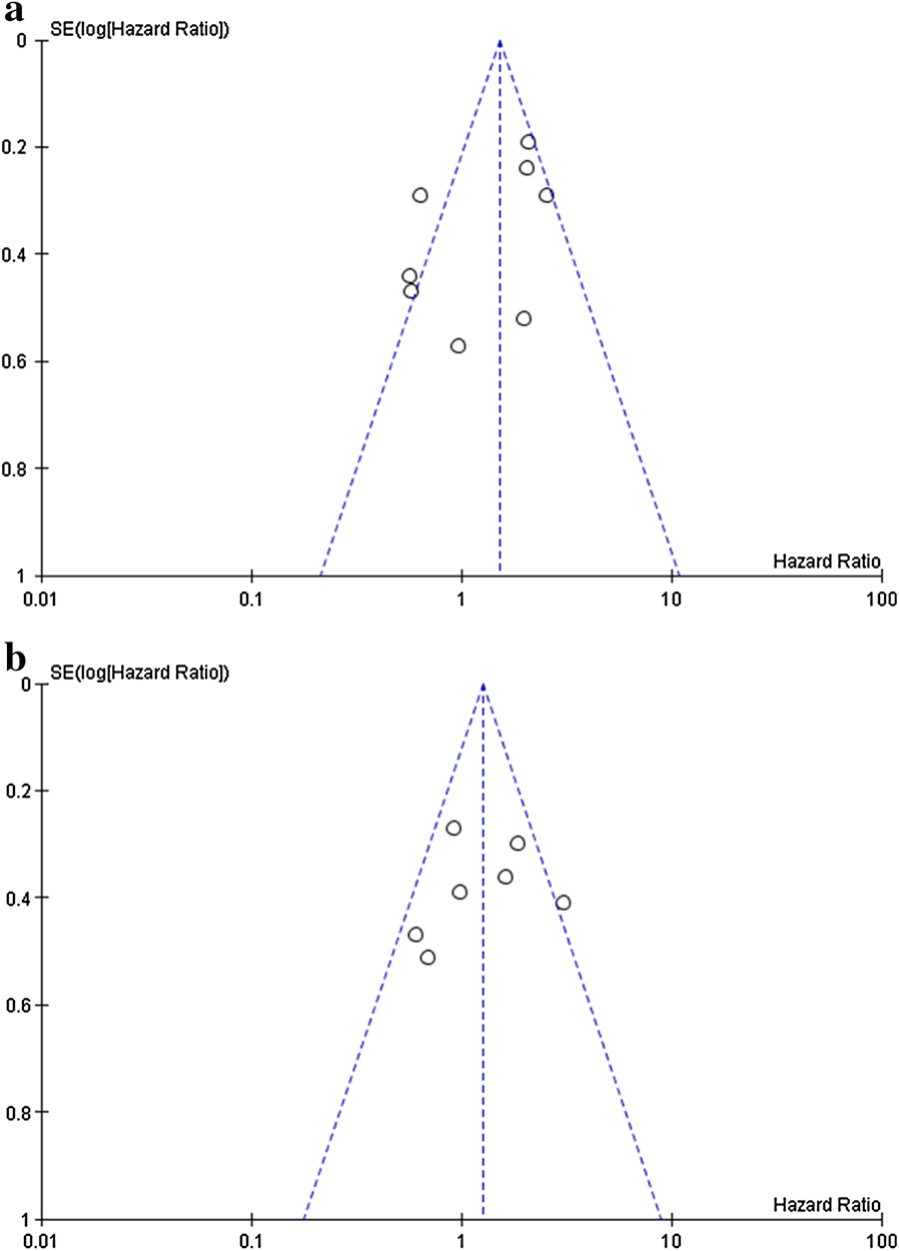
Funnel plot of hazard ratios for OS or DFS (**a**) and PFS or MFS (**b**). *Circles* in each plot represent individual studies.

The funnel plots of OS or DSS (Fig. 3a) and PFS or MFS (Fig. 3b) were almost symmetric, suggesting a low risk of publication bias.

## Discussion

Many studies have reported the prognostic impact of SS18–SSX fusion type on synovial sarcomas, but it still remains a matter of debate (Kawai et al. 1998; Ladanyi et al. 2002; Guillou et al. 2004; Skytting 2000; Nilsson et al. 1999; Hill et al. 2003; Canter et al. 2008; Panagopoulos et al. 2001; Mezzelani et al. 2001; Takenaka et al. 2008; ten Heuvel et al. 2009; Sun et al. 2009; Krieg et al. 2011; Charbonneau et al. 2013; Ren et al. 2013). One of the major problems with such studies is that many have limited sample power, analyzing only relatively small numbers of patients with synovial sarcoma. Therefore, we conducted meta-analysis to derive more robust estimates of predictive performance of SS18–SSX fusion gene, which to our knowledge had not been studied previously. The results of our meta-analysis using the data of a sufficient number of patients indicate that there was no significant difference in OS or DSS between patients with SS18–SSX1 and SS18–SSX2, but that there were indications of SS18–SSX1 being an unfavorable prognostic factor of PFS or MFS.

Our study was based on thorough literature searches and careful data extraction, and included a large number of patients after the methodological quality of study design assessments. The exclusion of studies with sample size smaller than 40 might contribute to the low risk of publication bias. However, our study still has several limitations. Firstly, follow-up periods are not long enough to evaluate survival outcomes of synovial sarcoma (Table 1). This is particularly important because metastases and local recurrence are known to develop very late in synovial sarcoma (Krieg et al. 2011). Secondly, bias could not be completely ruled out, despite our efforts to judge as fairly as possible. To minimize bias in the study selection and in the data extraction, reviewers performed this study blindly and independently. To ensure that all the selected articles were high-quality, only articles with six or more out of nine stars in the NOS assessment tool were selected. Thirdly, there was high and medium heterogeneity of HR across the studies of survival analyses. I^2^ represents the percentage of total variability in estimates caused by genuine between-study heterogeneity rather than by random sampling error, and was classified as follows: no heterogeneity (less than 25%), low heterogeneity (between 25 and 50%), medium heterogeneity (between 51 and 75%), and high heterogeneity (greater than 75%) (Higgins and Thompson 2002). In the current study, the observed heterogeneity might be attributable to the patient group comprising a very heterogeneous population where there was no agreement on surgical and adjuvant treatment modalities.

Thus far, there have been two large studies including more than 100 patients with primary localized synovial sarcoma reporting the prognostic impact of SS18–SSX fusion type (Ladanyi et al. 2002; Guillou et al. 2004). However, these results do not seem to be conclusive, since the follow-up periods of both studies are too short to evaluate the survival of slow growth malignancy. The only prognostic study evaluating a follow-up of survivors for a minimum of 10 years described that distant metastases occurred at a mean of 5–7 years and advocated that patients with synovial sarcoma should be tracked for more than 10 years (Krieg et al. 2011). Prospective randomized clinical trials with long follow-up cohorts using the same therapeutic strategy are ideal means of completely excluding all potential biases.

## Conclusions

In conclusion, the meta-analysis of this study has suggested that there was no significant difference in OS or DSS between patients with SS18–SSX1 and SS18–SSX2, but there were indications of SS18–SSX1 being an unfavorable prognostic factor of PFS or MFS. In order to consolidate our results, meta-analysis including cohorts with a follow-up period spanning at least 10 years will be necessary.

## Additional file

**Additional file 1.** Excluded and included articles after reviewing the complete article because of partially duplicated patients.
