# Supplementary material for: Prognostic value of SS18–SSX fusion type in synovial sarcoma; systematic review and meta-analysis
Source: Springerplus. 2015 Jul 25;4:375. doi: 10.1186/s40064-015-1168-3 (PMC4514732; doi:10.1186/s40064-015-1168-3)
Supplement: Supplementary file 1 — Additional file 1. Excluded and included articles after reviewing the complete article because of partially duplicated patients. [file 40064_2015_1168_MOESM1_ESM.docx]

**Supplemental Table** Excluded and included articles because of partially duplicated patients

| Reference | Year | Patient number | Institutes |
| --- | --- | --- | --- |
| Kawai^5^ (Excluded) | 1998 | 45 | MSKCC |
| Nilsson^14^ (Excluded) | 1999 | 34 | SSG |
| Canter^16^ (Excluded) | 2008 | 132 | MSKCC |
| Ladanyi^6^ (Included) | 2002 | 242 | MSKCC, SSG, and Others |

*MSKCC* Memorial Sloan-Kettering Cancer Center, SSG Scandinavian Sarcoma Group, *Others* including University of Nebraska Medical Center, Royal Marsden Hospital/Institute for Cancer Research, University of Pennsylvania Medical Center, Cleveland Clinic, and John Hopkins Hospital.
